# Precision medicine for locally advanced breast cancer: frontiers and challenges in Latin America

**DOI:** 10.3332/ecancer.2019.896

**Published:** 2019-01-22

**Authors:** Joseph A Pinto, César H Saravia, Claudio Flores, Jhajaira M Araujo, David Martínez, Luis J Schwarz, Alberto Casas, Leny Bravo, Jenny Zavaleta, Brigitte Chuima, Hober Alvarado, Ricardo Fujita, Henry L Gómez

**Affiliations:** 1 Unidad de Investigación Básica y Traslacional, Oncosalud-AUNA, Lima 15036, Perú; 2 Departamento de Radioterapia, Oncosalud-AUNA, Lima 15036, Perú; 3 Departamento de Medicina Oncológica, Oncosalud-AUNA, Lima 15036, Perú; 4 Escuela Profesional de Medicina Humana, Universidad Privada San Juan Bautista, Lima 15067, Perú; 5 Laboratorio Clínico, Clínica Delgado, Lima 15074, Perú; 6 Facultad de Ciencias Biológicas, Universidad Nacional San Luis Gonzaga de Ica, Ica 11004, Perú; 7 Centro de Genética y Biología Molecular, Universidad de San Martín de Porres, Lima 15024, Perú; 8 Departamento de Medicina Oncológica, Instituto Nacional de Enfermedades Neoplásicas, 15038, Perú

**Keywords:** locally advanced breast cancer, precision medicine, genomic platforms

## Abstract

Advances in high-throughput technologies and their involvement in the ‘omics’ of cancer have made possible the identification of hundreds of biomarkers and the development of predictive and prognostic platforms that model the management of cancer from evidence-based medicine to precision medicine. Latin America (LATAM) is a region characterised by fragmented healthcare, high rates of poverty and disparities to access to a basic standard of care not only for cancer but also for other complex diseases. Patients from the public setting cannot afford targeted therapy, the facilities offering genomic platforms are scarce and the use of high-precision radiotherapy is limited to few facilities. Despite the fact that LATAM oncologists are well-trained in the use of genomic platforms and constantly participate in genomic projects, a medical practice based in precision oncology is a great challenge and frequently limited to private practice. In breast cancer, we are waiting for the results of large basket trials to incorporate the detection of actionable mutations to select targeted treatments, in a similar way to the management of lung cancer. On the other hand and paradoxically, in the ‘one fit is not for all’ era, clinical and genomic studies continue grouping our patients under the single label ‘Latin American’ or ‘Hispanic’ despite the different ancestries and genomic backgrounds seen in the region. More regional cancer genomic initiatives and public availability of this data are needed in order to develop more precise oncology in locally advanced breast cancer.

## Introduction

Breast cancer is a highly prevalent malignancy causing ≈45,000 deaths every year in women in Latin America (LATAM) and the Caribbean [33]. The poorest outcomes of breast cancer seen in the region contrast with the lower mortality reported in developed countries. It is a product of late stages diagnosis and deficient cancer care [46].

There are several barriers leading to inadequate cancer management in LATAM, including lack of treatment coverage, lack of access to specialised cancer care and geographical barriers, among others [24]. If access to a standard of care in LATAM patients is complicated by a series of barriers previously exposed, the approach to precision medicine is challenging and mainly limited to private practice.

Advances in genomic profiling of breast cancer have led to a deep knowledge of the mechanisms of the disease and the identification of several actionable mutations. Latin American scientists participate actively in genomic research of several cancers while oncologists in the region are well-trained to integrate genomic data into patient management. Despite these advances, the lack of approved targeted treatments (other than mTOR inhibitors and anti-HER2 agents) makes the genomic profiling of breast cancer less attractive compared with other tumours, such as lung cancer.

In this manuscript, we reviewed the status of precision medicine in LATAM and its current use in the management of locally advanced breast cancer.

### Breast cancer subtypes

Since the year 2000, breast cancer has been revealed as a heterogeneous disease composed of at least four distinct molecular entities: luminal A, luminal B, HER2 and Basal-like tumours. Molecular classification of breast cancer is possible assessing four clusters of genes related to cell proliferation, Her2 signalling and luminal and basal epithelial-related genes [63, 76].

The molecular classification proposed by Perou and Sorlie was based in the evaluation of more than 500 transcripts; however, in 2009, Parker *et al* [59] proposed a set of 50 ‘intrinsic’ genes able to identify the breast cancer subtypes and with great prognostic capability. This molecular test was named PAM50 [59]. These subtypes can be inferred with immunohistochemical (IHC) assessment of oestrogen receptor, progesterone receptor and HER2 [18]. The marker Ki-67 improves the detection of luminal A tumours (these tumours present Ki-67 staining < 14%) [22]. Luminal A is defined by IHC by positivity to any hormone receptor (estrogen receptor (ER) or progesterone receptor (PR)) negativity to HER2 and a KI-67 staining in <14% of tumour cells; Luminal B is defined by positivity to any hormone receptor and a Ki-67 staining ≥ 14%; HER2 subtypes present negativity to both hormone receptors and positivity to HER2 and triple-negative breast cancers (TNBC) present negativity to both hormone receptors and HER2.

One typical feature of LATAM patients is a high prevalence of triple-negative breast cancer (up to 21.3%) in contrast to Caucasian populations (around 9%) [82, 86]. Subtypes identification is performed mainly by IHC in the clinical routine. The study of the epidemiology of breast cancer subtypes in LATAM is faced with heterogeneity between laboratories, antibodies and reagents used in the IHC, interpretation and most important, the assessment of Ki-67.

**Figure 1.**
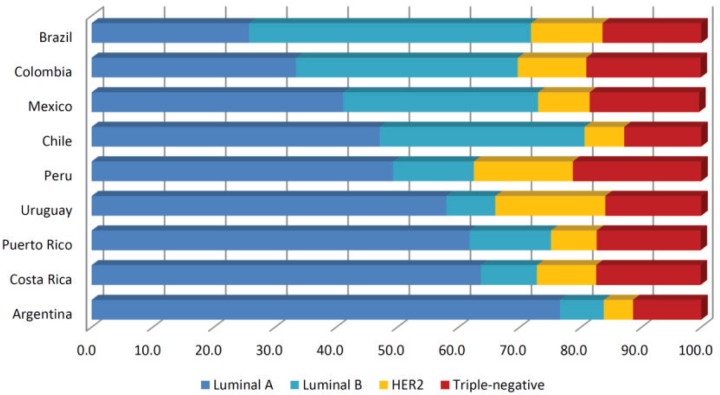
Distribution of breast cancer subtypes in Latin American countries (in percentages). We evaluated publications based on hospital registries. Papers with selected groups of patients were excluded (e.g. cohorts entirely composed of non-metastatic patients or operable patients). We obtained the average subtype’s composition when two or more studies were reported for the same country. Papers selected were: [1, 3, 12, 16, 20, 27, 54, 57, 61, 62, 66, 72, 77 and 83].

Regarding to the prevalence of breast cancer subtypes, distribution of HER2 and triple-negative subtypes is the most homogeneous among LATAM countries (ranging from 4.8%–18% and 13%–21.3%, respectively), contrasting to frequencies of luminal A (25.8%–76.6%) and luminal B tumours (7.2%–46.1%) (Figure 1). Heterogeneity seen in luminal tumours could be attributed to the difficulty to discriminate luminal A from luminal B cases, where Ki-67 staining plays a major role. Yabar *et al* [86] evaluating a mixed cohort of patients from Peru and Uruguay, found that distribution of luminal A tumours change from 41.1% to 31.9% after the inclusion of Ki-67 in the IHC panel to determine the breast cancer subtypes. Inclusion of Ki-67 in the IHC panel to evaluate recently diagnosed breast tumours is relatively new and therefore in the coming years, reports about the distribution of breast cancer subtypes in LATAM will be more accurate and less heterogeneous.

## Mutational profile of breast cancer, genomic platforms and opportunities to precision medicine

The breast cancer genome has been deeply studied and there are several initiatives in malignancy that have revealed the mechanism of disease, mechanisms of drug resistance and potential drug targets. In addition, particular mutational patterns in the breast cancer subtypes have been found, for example, high frequencies of mutations in the PI3K/AKT pathway in luminal A tumours (>40% of cases) and high rates of *TP53* mutations in HER2 and basal-like tumours (>70%), as shown in large genomic projects [10, 17, 60]. The public availability of these data is boosting the research of this malignancy around the globe.

Some regional initiatives have shown some molecular features of breast cancer in LATAM. In regard to mutations in *PIK3CA*, mutations were reported in 27% of breast cancer cases in Brazil and in 15.7% of HER2-amplified and triple negative non-metastatic cases in Peru [21, 51]. TP53 was also studied in LATAM. In Brazil, frequency mutations in this gene are reported in 73.3% of patients, while the founder mutation *TP53* p.R337H is detected in 0.3% of the general population in southern Brazil and it is responsible for 3.4–8.6% hereditary or familiar breast cancers [51, 90]. In a cohort of Peruvian patients with triple-negative breast cancer, 89% of cases bearing TP53 mutations were described [9]. In this group of patients, JAK2 and MYC amplification (12.5% and 35%, respectively) were recently described as alterations with the potential to be targeted to small molecules inhibitors and were statistically related with shorter survival [8, 9].

A review of the most frequent mutations in breast cancer points to the potential incorporation of targeted treatments, in addition to the currently approved drugs in a high proportion of patients (Table 1).

Nowadays, there is a wide availability of genomic platforms offered to cancer profiling. Undoubtedly, the most successful model of the incorporation of genomic platforms is in the management of non-small cancer lung cancer. Regarding breast cancer, multigene assays to calculate risk scores, including, OncotypeDx, Mammaprint, Prosigna, Endopredict and others, have gained a high value and wide acceptance in the clinical routine in early breast cancer at low risk [48].

Despite that fact that in LATAM the cost of adjuvant treatment is cheaper than the cost of these platforms to justify a cost-benefit of avoidance of chemotherapy, patient-centred reasons, such as avoidance of unwanted toxicities and better quality of life, have led to the incorporation of these molecular tests in the coverage of private insurance. Oncosalud, the largest pre-paid system in Peru, offers for-free testing with OncotypeDX for their affiliates bearing early-stage hormonal receptor-positive breast tumours at low risk.

Despite several studies conducted in locally advanced breast tumours, there is not a clear indication of molecular testing to select the therapy or to stratify risks in this setting. The MD Anderson Cancer Center conducted a study in LABC, in which Latin American Institutions participated with the intention of developing a genomic predictor (based in cDNA microarrays) of response to neoadjuvant paclitaxel and 5-fluorouracil, doxorubicin and cyclophosphamide [42, 91]. Another Latin American initiative included the development of a three-gene signature based on the expression of *CCL5*, *DDIT4* and *POLR1C* assessed in residual disease and able to stratify patients in two risk groups and predict the outcome of patients with triple negative breast cancer resistant to neoadjuvant chemotherapy [64]. This study led to other works that improved the understanding of the biology of *CCL5* and *DDIT4* and contributed to the evaluation of other biomarkers in cancer [5, 26, 35, 65].

## Hereditary breast cancer

Several efforts have been made in Latin American countries to determine the proportion of breast cancer patients bearing germline mutations (Table 2). In unselected patients, BRCA1 and BRCA2 mutations are reported in ranges of 0.3%–13% and 0.75–7.41%, respectively, while in high-risk/hereditary/familiar breast cancer patients, mutations in *BRCA1* and *BRCA2* were reported in 0.9%–16.6% and 0.49%–18.2%, respectively (Table 2).

Frequencies of mutations and the finding of new variants could change according to the technology of sequencing and extension of the gene that will be evaluated (e.g. hotspots versus the entire gene). The work of Vaca-Paniagua *et al* [81] using pyrosequencing determined a frequency of 41% of BRCA genes alterations in Mexican patients, where 10% were known mutations while the rest were novel or variants with unknown clinical significance. On the other hand, Buleje *et al* [14] in a comprehensive evaluation of *BRCA* genes, reported a new mutation in the Peruvian population. It indicates the great need for comprehensive profiling of germline mutations in LATAM with the intention to develop more accurate tests, which are customised for this population.

Other genes linked to a high susceptibility described in LATAM patients includes *ATM, BARD1, CHECK2, FGFR2, GSTM1, MAP3K1, MTHFR, PALB2, RAD51, TOX3, TP53, XRCC1 and 2q35* [45]*.*

There are several founder mutations identified in LATAM, including *BRCA1* del exons 9–12, in Mexico; *BRCA1* 5382insC and *BRCA2* c.156_157insAlu in Brazil and *BRCA1* 3450del4, A1708E and BRCA2 3034del4, in Colombia [58]. A worldwide study evaluating 29,700 families showed different mutational patterns in BRCA genes in the Latin American/Hispanic population, characterised by fewer mutations in the BRCA1 C-terminal domain and a higher prevalence of the mutation c.3264dup in *BRCA2,* in contrast to alterations observed in African American and Asian individuals [68].

We need to improve the access to a molecular screening of germline mutations in the region not only for people at high risk of breast and ovarian cancer but also for individuals with other medical conditions.

## Potential role of liquid biopsy in breast cancer

The term liquid biopsy is used to define a sample obtained from biological fluids, such as blood, cerebrospinal fluid, semen and others with the aim to detect and evaluate circulating tumour cells (CTCs), circulating-free DNA (cfDNA), exosomes and other molecules. The study of liquid biopsies has had a great impact in the management of non-small cell lung cancer (NSCLC), which is indicated in cases where a sample of the surgical specimen or a tumour biopsy is not available or insufficient or in patients with progressive or recurrent disease during treatment with tyrosine kinase inhibitors [70].

In breast cancer, liquid biopsies have great potential to be incorporated in early diagnosis of this disease. Although in this setting, tests evaluating counts of mutant cfDNA fragments have low detection rates (33%) [11], counting copy numbers of specific genes could be more useful as a diagnostic test. In a case-control study, breast cancer patients had a higher number of cfDNA copies of *PUM1* and *RNaseP* genes detected in peripheral blood compared with healthy controls. The median count for *PUM1* was 4.6 copies/μL (standard deviation [SD]: 1.96) versus 10.6 copies/μL (SD: 3.82) and for *RNaseP,* 3.2 copies/μL (SD:1.57) versus 7.6 copies/μL (SD:3.45) for healthy controls versus breast cancer patients, respectively (*p* <0.0001 in both cases) [6].

In the advanced setting (locally advanced/metastatic), 86% of cases present detectable circulating tumour DNA in the blood [56]. A recent study evaluating 21,807 advanced cancers, in which 16% were breast tumours, showed the utility of the liquid biopsy to detect targetable mutations in breast cancer patients, for example, alterations in *PIK3CA, ESR1* and *MYC*, *FGFR1, HER2* amplifications [89].

Regarding the CTCs count in LABC, it seems not to be related with the response to neoadjuvant chemotherapy [32]; however, in metastatic breast cancer, CTCs provides prognostic information. Detection of ≥5 CTCs in peripheral blood confers a worse prognosis in terms of disease-free survival (HR = 2.34; *P* = 0.0016) or overall survival (OS) (HR = 3.56; *P* = 0.0044) [25].

In contrast with cfDNA and CTCs, the prognostic value of exosomes has not been defined in breast cancer although it has a well-established role in the biology of breast cancer.

The use of liquid biopsies to direct targeted therapy in breast cancer, as well as in other malignant tumours, will depend on the results of large basket trials such as the National Cancer Institute’s Molecular Analysis for Therapy Choice, and others, if they demonstrate that treating a targetable mutation in breast cancer is translated to benefit in outcomes.

Despite the fact that the study of liquid biopsies can aid in appropriate patient stratification for targeted therapy and provide important prognostic information, its incorporation in the clinical routine in LATAM, nowadays, is limited.

## Immunotherapy in breast cancer

The value of immunotherapy in breast cancer is not yet well established as is the case for lung cancer, melanoma and other malignancies. The heterogeneity of breast tumours is in part responsible, while the future success of immunotherapy depends on the ability of emerging biomarkers to identify patients who will benefit from the therapy [41].

TNBC cases exhibit a different immunological profile to other breast cancer subtypes with an enriched inflammatory signature and a high expression of the Human Leukocyte Antigen [50]. These characteristics confer to TNBC more opportunities to obtain benefits from immunotherapy in contrast with luminal tumours while HER2 tumours also show potential to get benefit from these drugs [55]. Up to date, one of the better results with an immune checkpoint inhibitor in breast cancer was achieved with atezolizumab in metastatic triple negative breast cancer with a median duration of response of 21 months [29].

On the other hand, in a remarkable study, Zacharakis *et al* [88] reported the case of a patient treated with tumour-infiltrating lymphocytes (reactive against mutations of the proteins SLC3A2, KIAA0368, CADPS2 and CTSB) plus interleukin-2 and pembrolizumab, achieving the complete durable regression of hormone-sensitive metastatic breast cancer [88].

Unfortunately, there is a lack of immunotherapy studies in our region. Large trials evaluating PD-1/PDL-1 inhibitors in breast cancer are not being conducted in LATAM. Up to date, only one immunotherapy study is active (source: clinicatrials.gov), whose purpose is to evaluate the immunogenicity and safety of dendritic cells, and which is recruiting patients in Colombia (NCT03450044).

## High-precision radiotherapy

The role of radiotherapy (RT) in the management of breast cancer has evolved significantly over the last decades. The addition of RT to conservation surgery significantly reduces the local recurrence rates which translate into a 5% decrease in breast cancer-specific cancer mortality to 15 years, regardless of lymph node status [92].

The treatment of LABC represents a challenge for radiation oncologists. Despite advances in surgery and systematic therapies, these patients continue to have a high risk of loco-regional recurrences which can lead to severe symptoms, compromised quality of life and distant metastasis. In locally advanced disease, radiation therapy to the chest wall and lymph node stations (supraclavicular, infraclavicular, axillary and internal mammary) associated with post-mastectomy chemotherapy reduces loco-regional recurrences and improves recurrence-free survival and OS. In addition, high-risk patients with large tumours, high histological grade and extensive lymph nodes involvement with radical axillary dissection benefit most from post-mastectomy RT [93–96].

The concept of personalised RT is not new but has evolved in parallel with advances in the identification of the target and the precise administration of the treatment; the technical aspects associated with the inclusion of tumour characteristics in the decision-making process can maximise the risk/ratio in this clinical scenario that requires individualisation and adaptation of treatments.

Innovative radiation delivery techniques such as intensity-modulated radiotherapy, volumetric arc therapy and administered under image-guided radiotherapy have improved the planning and administration of RT to the tumour while minimising the dose delivered to healthy tissues. These techniques not only deliver the radiation with high precision but also diminish the treatment times allowing it to be performed as fast as 1.5 minutes on beam time (Pierce *et al*, 2002). In addition, in oligometastatic, oligoprogressive or oligorecurrent disease, RT offers highly precise techniques, including intracranial stereotactic radiosurgery and fractionated stereotactic radiation therapy and stereotactic ablative RT or stereotactic body radiation therapy [97].

From the perspective of the radiation oncologist, the spectrum of locally advanced cancer management changes rapidly. The broad understanding of the biological complexities of breast cancer has led to better systemic therapies and targeted therapies which are increasingly available. It is imperative that advanced RT treatments go hand in hand with these efforts in order to provide personalised therapies and improve outcomes in this scenario which is a challenge for treatment.

Also, in the Immuno-oncology era, there are multiples studies trying to address the best combination of target therapy/chemotherapy and immunotherapy in locally advanced and metastatic breast cancer, not all metastatic sites are created equal, most tumours are not innately responsive to checkpoint blockade alone—we must look for immunogenic neoantigen presentation, tumour microenvironment and better patient selection/host features to see how they could interact with each other in order to improve desired outcomes [87].

In the era of personalised RT for the treatment of locally advanced breast cancer, radiation treatments must be adapted to the individual risks of each patient, as well as the intrinsic biology of the tumours.

### Precision medicine in medical education

The World Federation for Medical Education defines medical education as ‘a continuum that starts with undergraduate training, continues with postgraduate training and extends with continuing education’, with the main objective of ‘training professionals with capacity to improve the health of the population’. Levels and contents of Medical Education must be permanently renewed, to adapt to changes, the hyper obsolescence of knowledge, the need to continually change medical knowledge and professional practice [47]. On the other hand, technology has undergone rapid evolution since the remodelling of the landscape of biomedical sciences two decades ago.

Clinical practice has evolved in this way from evidence-based medicine to a custom-tailored therapeutic approach. Nowadays, patients are well informed and have wide access to the latest scientific information, clinical practice guidelines and also to direct-to-consumers genetic tests. Introduction of precision medicine in the medical school curriculum is essential.

To improve the education of physicians prepared for the age of genomic medicine, it is suggested that reforms should be made to the medical curriculum, including making genetics a core competency (like anatomy) and introducing students to computational methods for genomic analysis [53]. In Peru, this necessity has been addressed since 2017 by the ASPEFAM, an association comprised of 27 medical schools from public or private universities.

Regional initiatives for higher education such as the Tuning LATAM project [40] should address the necessity to improve the competencies in precision medicine for undergraduate students at a high standard in order to obtain highly competitive health professionals.

## Challenges for precision medicine in the region: ‘the Latin American Genoma’

The term ‘LATAM’ was coined in the middle of the 19th century with the geopolitical purpose to designate the territories of the Americas with French, Spanish and Portuguese colonies [67]. Despite the great differences in cultural, ethnic and genetic backgrounds in LATAM, for genomic or clinical research purposes, the single term ‘Latin American’ or ‘Hispanic’ is commonly used to designate people living in or migrating from countries in this region.

Several studies have shown different genetic structure according to the geographical distribution in LATAM [85], while in the same country it is possible to observe population groups with great genetic differences. This is the case for Peru, despite the fact that the majority of the population lives on the coast, mostly mestizos; there are several communities in the Andes and in the jungle, with a divergent genetic background, explained by the isolation [71]. Particularities in the genetics of the Peruvian population could explain, for example, higher rates of EGFR mutations seen in NSCLC compared with other Latin American countries (believed to be due to a higher proportion of Asian ancestry in Peru compared with other LATAM countries) [65, 75].

This diverse genetic background has a practical relevance into the interpretation of cancer genome sequencing because of the presence of germline variants in the population, which could be confused with somatic activating mutations of the tumour. The Latin American population has a higher distribution of unique missense variants compared with the European population [39].

Environmental factors could shape the nature of particular cancer and this is responsible for the different epidemiology seen in some cancers in the region. Well-known examples of this environment-epidemiology relationship correspond to the highest incidence of gallbladder cancer in Chile compared to other countries in the region [7]. In addition, Hepatocellular carcinoma in Peru has different epidemiology (earlier onset and related to hepatitis B virus infection) and molecular patterns in contrast with other countries within this region or the world [52].

Regarding breast cancer, in LATAM, there is a higher prevalence of TNBC compared with Caucasian populations [83]. However, it is suggested that the proportion of TNBC which is related to socioeconomic conditions [13], genetic or environmental explanations needs to be explored.

There is a great need for a better genomic characterisation of different cancers in the region at country level and with publicly available data in order to improve the genomic tools for a better risk-stratification and a more precise therapy selection in our LATAM patients.

## Future perspectives and conclusion

‘Latin American’ or ‘Hispanic’ are convenient terms used commonly in clinical or genomic research; however, people with different ancestry and genetic background are grouped under this label. In order to improve our approach for precision medicine in cancer, regional genomic initiatives should be initiated and empowered. Although precision medicine in LATAM is often limited to private practice, there is a huge challenge to be faced in making it more inclusive to benefit patients.

## Figures and Tables

**Table 1.** Top 15 altered genes described in several breast cancer genomic projects (data retrieved from cbioportal.org).

Metabric *N* = 2509		British Columbia (*N* = 65)		Broad Institute Samples *N* = 103		Sanger Institute *N* = 100		TCGA Provisional (*N* = 1105)		Mutational Profile of Metastatic Breast Cancers Samples *N* = 216		The Mestastatic Breast Cancer Project *N* = 157	
Gene	%	Gene	%	Gene	%	Gene	%	Gene	%	Gene	%	Gene	%
*PIK3CA*	41.1	*TP53*	53.80	*PIK3CA*	27.1	*TP53*	37	*PIK3CA*	32.1	*TP53*	34.7	*TP53*	33.1
*TP53*	36.4	*PIK3CA*	10.7	*TP53*	27.1	*PIK3CA*	30	*TP53*	30.3	*PIK3CA*	29.5	*PIK3CA*	32.4
*MUC16*	17.2	*MYO3A*	9.2	*TTN*	11.6	*TTN*	26	*TTN*	15.8	*TTN*	16.9	*TTN*	18.4
*AHNAK2*	16.6	*USH2A*	9.2	*KMT2C*	6.8	*GATA3*	15	*CDH1*	11.2	*ESR1*	9.8	*CDH1*	14.0
*SYNE1*	12.4	*PTEN*	7.6	*MUC2*	5.8	*KMT2C*	11	*GATA3*	9.7	*RYR2*	9.3	*HMCN1*	10.8
*KMT2C*	11.8	*ADGRG4*	6.1	*AKT1*	5.8	*MUC16*	10	*MUC16*	7.4	*GATA3*	8.9	*MUC16*	10.8
*GATA3*	11.3	*ATR*	6.1	*FLG*	5.8	* CDH1*	8	*MAP3K1*	7.1	*HMCN1*	7.0	*PTEN*	9.5
*CDH1*	9.9	*ZNF142*	6.1	*MUC16*	5.8	*BIRC6*	7.0	*KMT2C*	7.0	*FRAS1*	7.0	*CSMD3*	8.9
*MAP3K1*	9.9	*UBR5*	6.1	*DMD*	4.8	*FSIP2*	7.0	*MUC12*	5.4	*USH2A*	7.0	*RYR1*	8.2
*DNAH11*	9.5	*LRP2*	6.1	*RYR2*	3.8	*SYNE1*	7.0	*MUC4*	5.3	*MUC16*	6.5	*KMT2C*	7.6
*AHNAK*	9.2	*TTN*	6.1	*RYR3*	3.8	*STAB2*	6	*FLG*	4.5	*RYR3*	6.5	*ASTN1*	7.0%
*DNAH2*	7.8	*COL6A3*	6.1	*AGRN*	3.8	*NCOR1*	6	*USH2A*	4.5	*DNAH6*	6.1	*DMD*	7.0
*KMT2D*	7.4	*RB1*	6.1	*SRRM2*	3.8	*DST*	6	*SYNE1*	4.3	*OBSCN*	6.1	*DNAH5*	7.0
*USH2A*	7.1	*SYNE1*	6.1	*ADAMTS7*	3.8	*CSMD1*	6	*RYR2*	4.2	*PKHD1L1*	6.1	*HUWE1*	7.0
*DNAH5*	7.0	*MDN1*	6.1	*HMCN1*	3.8	*MAP3K1*	6	*NCOR1*	4.0	*MAP3K1*	6.1	*OBSCN*	7.0
	**Prognostic Value**												
	**Targetable mutation**												

**Table 2.** Studies in Latin American Countries evaluating mutations in BRCA genes.

Country	Cohort (*N*)	Population characteristics	Número de pacientes con mutaciones		% patients with mutations		References
			BRCA1	BRCA2	BRCA1	BRCA2	
Argentina	134	97 Family history + 37 unselected	23	15	17.16	11.19	Solano *et al* [73]*
Argentina	940	Family history	105	74	11.17	7.87	Solano *et al* [74]*
Brazil	402	Unselected	6	3	1.49	0.75	Gomes MC *et al* [36]
Brazil	612	Family history	18	3	2.94	0.49	Esteves *et al* [30]
Brazil	54	Unselected	7	4	12.96	7.41	Carraro *et al* [19]
Chile	54 fam^+^	Family history	4	7	7.14	12.96	M A Gallardo *et al* [34]*
Chile	64 fam^+^	Family history	7	3	10.94	4.69	Jara L. *et al* [44]*
Chile	326 fam^+^	High risk	11	12	3.37	3.68	Gonzalez-Hormazabal *et al* [37]
Chile	453	336 Family history 117 High risk	32	39	7.06	8.61	Álvarez *et al* [4]
Colombia	53 fam^+^	Family history	8	5	15.09	9.43	Torres *et al* [79]
Colombia	244	Unselected	2	1	1.2	0.36	Hernández *et al* [43]
Colombia	85	Family history	7	8	8.24	9.41	Cock-Rada *et al* [23]
Costa Rica	111	Family history	1	4	0.9	3.6	Gutiérrez Espeleta *et al* [38]
Cuba	307	Unselected	1	7	0.33	2.28	Rodríguez *et al* [69]
Mexico	22	Early onset breast cancer	2	4	9.09	18.18	Calderon-Garcidueñas *et al* [15]*
Mexico	39	Family history	16	10	41.03	25.64	Vaca-Paniagua *et al* [81]*
Mexico	810	Unselected	20	14	2.47	1.73	Torres-Mejía *et al* [80]*
Mexico	96	Unselected	11	3	11.46	3.13	Villarreal Garza *et al* [84]
Peru	18 fam^+^	Family history	3	1	16.67	5.56	Buleje *et al* [14]
Peru	266	Unselected	11	2	4.14	0.75	Abugattas *et al* [2]*
Uruguay	42	Family history	2	5	4.76	11.90	Delgado *et al* [28]
Venezuela	58	High risk	6	4	10.34	6.90	Lara *et al* [49]

* Includes novel, possible damaging and/or variants of unknown significance

+ Fam= Families

HBOC= hereditary breast and ovarian cancer
